# Research on an Autonomous Localization Method for Trains Based on Pulse Observation in a Tunnel Environment

**DOI:** 10.3390/s24175556

**Published:** 2024-08-28

**Authors:** Jianqiang Shi, Youpeng Zhang, Guangwu Chen, Yongbo Si

**Affiliations:** School of Automation and Electrical Engineering, Lanzhou Jiaotong University, Lanzhou 730070, China; t424114331@163.com (J.S.);

**Keywords:** pulse observation, tunnel environment, combined positioning model, EM algorithm

## Abstract

China’s rail transit system is developing rapidly, but achieving seamless high-precision localization of trains throughout the entire route in closed environments such as tunnels and culverts still faces significant challenges. Traditional localization technologies cannot meet current demands, and the present paper proposes an autonomous localization method for trains based on pulse observation in a tunnel environment. First, the Letts criterion is used to eliminate abnormal gyro data, the CEEMDAN method is employed for signal decomposition, and the decomposed signals are classified using the continuous mean square error and norm method. Noise reduction is performed using forward linear filtering and dynamic threshold filtering, respectively, maximizing the retention of its effective signal components. A SINS/OD integrated localization model is established, and an observation equation is constructed based on velocity matching, resulting in an 18-dimensional complex state space model. Finally, the EM algorithm is used to address Non-Line-Of-Sight and multipath effect errors. The optimized model is then applied in the Kalman filter to better adapt to the system’s observation conditions. By dynamically adjusting the noise covariance, the localization system can continue to maintain continuous high-precision position information output in a tunnel environment.

## 1. Introduction

With the rapid development of rail transit, the importance of train localization technology has become increasingly prominent. A continuous and reliable high-precision train positioning system is crucial for ensuring operational safety and improving transportation efficiency. However, in enclosed environments such as tunnels, traditional satellite positioning technology cannot work effectively due to satellite signals being blocked or attenuated, which poses significant challenges for train positioning [1]. To overcome the challenges of train positioning in the tunnel environment, researchers have been exploring new positioning methods [2,3].

In recent years, the positioning technology based on multi-sensor fusion has attracted much attention due to its numerous advantages. It can overcome the limitations of traditional positioning technologies in a tunnel environment, enhancing the accuracy, continuity, and reliability of train positioning, while providing strong support for automatic train operation and intelligent scheduling [4]. Currently, commonly used sensors in integrated navigation and positioning systems include inertial modules, pulse odometers, and visual sensors, which are conducive to overcoming the limitations of a single sensor in complex environments. For example, in enclosed environments such as tunnels, satellite signals are highly susceptible to being blocked or attenuated; additionally, the lack of redundancy and fault tolerance mechanisms mean that if the sensor fails or malfunctions, the entire positioning system will cease to function properly [5]. Among these, common noise reduction methods for inertial modules include Wiener filtering, Kalman filtering, and Wavelet threshold denoising [6]. The denoising method proposed by Zhou et al. combines BP neural network and Kalman filtering and requires prior information and state equations, making the solving process complex [7]. Dong et al. improved accuracy by improving wavelet bases, decomposition levels, and threshold functions. However, the wavelet denoising makes it difficult to choose the optimal threshold function [8]. The wavelet denoising method proposed by Wang et al. under the strong noise conditions faces issues such as difficulty in selecting the wavelet bases, the need for prior determination, and poor adaptability [9]. The method based on Empirical Mode Decomposition (EMD) was proposed by Huang et al. in 1998, and it is a novel signal decomposition method suitable for the noise reduction processing of nonlinear and non-stationary signals [10]. In train positioning systems, sensor signals often contain a significant amount of noise and interference, requiring effective noise reduction and decomposition techniques to extract useful information. EMD has been widely applied to eliminate random errors in MEMS gyroscope [11,12,13], and due to the issue of mode mixing in EMD, several enhanced methods have been proposed, including Ensemble Empirical Mode Decomposition (EEMD), Complementary Ensemble Empirical Mode Decomposition (CEEMD), and Adaptive Noise Empirical Mode Decomposition (ANEMD). These methods aim to address the challenges associated with mode mixing and improve the performance of EMD in various applications. The signal decomposition effect of the EMD method is closely related to the envelope fitting effect, and outliers have a significant impact on the envelope fitting effect of the cubic spline method based on extreme points. Therefore, in noise reduction processes, directing the application of EMD for denoising typically yields moderate results [14]. Chen et al. studied a denoising method that combines Energy Ratio (ER) with EMD; the method distinguishes noise intensity based on gyroscope drift signals but does not proposes a clear criterion for selecting intrinsic mode functions (IMFs) [15]. Li et al. used EMD followed by a Kalman filter for gyroscope error compensation with good results, but the issue of mode aliasing was not resolved [16].

Comparing with existing autonomous integrated positioning algorithms, in complex and variable environments, traditional fixed-parameter filtering algorithms struggle to meet the demands of high-precision positioning. Adaptive filtering algorithms, on the other hand, can dynamically adjust parameters based on environmental changes, thereby enhancing filtering performance. This work highlights the following three contributions. Firstly, outlier gyro values are removed according to the Letts criterion to improve the envelope fitting effectiveness of EMD. Subsequently, the preprocessed gyro data undergo signal decomposition using Complementary Ensemble Empirical Mode Decomposition with Adaptive Noise (CEEMDAN). The decomposed IMFs are partitioned using methods such as continuous mean square error and norm criteria. Next, forward linear filtering and dynamic threshold filtering are applied separately to the IMF components containing varying degrees of valid signal components for denoising. This achieves gyro denoising while maximizing the effective components in the reconstructed gyro signal.

Secondly, the accuracy of the observation model has a direct impact on the performance of the positioning system. In complex environments, it is necessary to establish a more sophisticated observation model to cope with various error sources. A thorough analysis of error sources is conducted, considering observational equations that incorporate installation errors and lever arm errors. Additionally, the odometer pulse equations are integrated into the measurement equations, enabling a detailed analysis of velocity components in inertial navigation systems. The modeling approach described above comprehensively analyzes the dynamic characteristics within the system, laying the groundwork for subsequent filtering algorithms to provide state estimation.

Finally, the EM algorithm is integrated with a Kalman filter to achieve an adaptive estimation of the covariance matrices for process noise and measurement noise. By utilizing the EM algorithm to compute expectations and maximize the log-likelihood function after prediction and update steps. This method dynamically adjusts noise parameters, which enhances the filter’s accuracy and robustness when handling systems with unknown or varying noise characteristics, making it suitable for complex dynamic environments.

## 2. Gyroscopic Signal Processing

In a typical integrated navigation system, the inertial measurement unit consists of accelerometers and gyroscopes. The measurement accuracy of gyroscopes is closely related to the system’s deterministic and random errors. Deterministic errors are typically compensated using system calibration methods, while random errors exhibit strong nonlinear and uncertain characteristics. These significantly affect mid- to low-grade gyroscopes, requiring more targeted approaches such as signal filtering and noise reduction techniques [17].

### 2.1. Basic Principle of Gyroscopic Noise Reduction

The conventional EMD method utilizes the local characteristic time scale of gyroscope signals to decompose them into a series of oscillating IMF components from high frequency to low frequency [18]. Based on the frequency characteristics of the IMFs, they are distinguished from high to low. In the signal denoising processing, where the characteristics of random noise are difficult to determine, high-frequency signals are often directly removed, and the recombination of low-frequency IMFs are treated as the effective signal. The noisy gyroscope signal *x*(*t*) is decomposed into *L* IMFs, which include modal components *h_i_*(*t*)(1 ≤ *I* ≤ *L*) and a remainder term *r_L_*(*t*). Here, *t* represents the corresponding time point for the given amplitude. The reconstructed signal of the gyroscope can be represented as follows:

(1) $$x\left(t\right)=\sum_{i=1}^{L}h_{i}\left(t\right)+r_{L}(t)$$

There are two issues in the entire signal processing process. Firstly, the traditional EMD may encounter mode mixing problems; secondly, the EMD method effectively decomposes signals, but it lacks clear criteria and guidelines for defining and separating IMFs that contain noise. CEEMDAN is an improved algorithm based on EEMD; it effectively reduces the number of iterations and addresses the mode mixing phenomenon, enhancing reconstruction accuracy. Its solution steps are as follows:

(1) In the preprocessed gyroscope signal, add the *I* set of random noise for *I* sets of sequences, each with a mean of 0 and a standard deviation of *β*_0_. Here, the *i* set sequence is *x*(*n*) *+ β*_0_*ω_i_*(*n*), where *ω_i_*(*n*) represents random white noise that follows a standard normal distribution. Upon decomposing the sequences with added noise using EMD, the first-order IMF satisfies the following:
  (2) $$V_{IMF}^{1}=\frac{1}{I}\sum_{i=1}^{1}{\left(V_{IMF}^{1}\right)}^{i}$$
(2) The residual error and the second-order IMF, respectively, satisfy the following:
  (3) $$r_{1}\left(n\right)=x\left(n\right)-V_{IMF}^{1}$$
  (4) $$V_{IMF}^{2}=\frac{1}{I}\sum_{i=1}^{1}(EMD\left(r_{1}\left(n\right)+\beta_{1}EMD\left(\omega_{i}\left(n\right))\right)\right)$$
(3) Following this recursive process, the *k* order residual error and the *k +* 1 order IMF, respectively, satisfy the following:
  (5) $$r_{k}\left(n\right)=r_{k-1}\left(n\right)-V_{IMF}^{k}$$
  (6) $$V_{IMF}^{k+1}=\frac{1}{I}\sum_{i=1}^{1}(EMD\left(r_{k}\left(n\right)+\beta_{k}EMD\left(\omega_{i}\left(n\right))\right)\right)$$
(4) Repeat the above process until *r_k_* satisfies the termination condition, and thus the trend term satisfies the following equation:
  (7) $$r\left(n\right)=x\left(n\right)-\sum_{K=1}^{K}V_{IMF}^{k}$$

After CEEMDAN decomposition, the signal *x*(*n*) is decomposed into several IMFs and a trend term, satisfying the following equation:

(8) $$x\left(n\right)=\sum_{K=1}^{K}V_{IMF}^{k}+r(n)$$

The above equations describe an iterative process. Here, *n* represents the iterative variable in the algorithm steps. The selection criteria for the iterative variable are typically based on termination conditions, such as reaching a predetermined number of IMFs or the maximum number of iterations.

In this paper, the improvement of the hybrid filtering structure is mainly reflected in the processing of gyroscope signals, especially in the refinement and adaptive adjustment in the process of signal decomposition and noise reduction. The Letts criterion is adopted to eliminate outliers in gyroscope signals, thereby improving the accuracy and stability of subsequent signal decomposition. The preprocessed gyroscope signals are decomposed using CEEMDAN, which can effectively reduce the phenomenon of modal aliasing and further improve the accuracy of signal decomposition.

### 2.2. Improved Hybrid Filtering Based on the Letts Criterion

The CEEMDAN algorithm decomposes the gyroscope signals into a series of IMFs, where low-order noise IMFs are directly removed. The first-order IMF is used to determine the threshold for hybrid intrinsic mode function filtering. However, when the carrier performs highly maneuverable movements, the low-order noise that causes the decomposition of the gyroscope signal is mixed with the high-frequency signal in the IMFs. Directly removing this noise between the IMFs can lead to the loss of high-frequency effective signals and cause the filter threshold to rise, resulting in excessive filtering of the hybrid IMFs.

**Figure 1:**
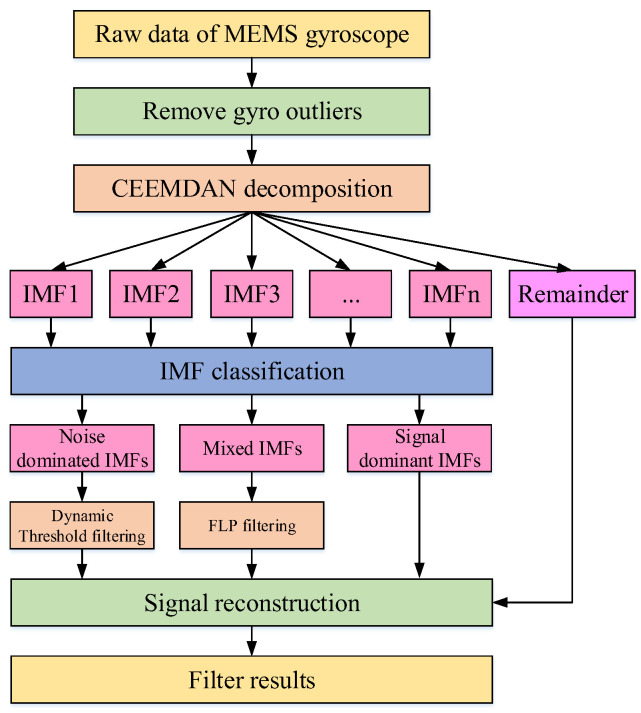
Flow chart of the improved hybrid filtering structure.

The principle of the improved hybrid filtering algorithm is shown in Figure 1. Firstly, the gyro’s raw data is processed to remove outliers using the Letts criterion. Then, the preprocessed gyroscope data undergoes CEEMDAN decomposition, resulting in a series of narrowband IMFs signals with frequencies ranging from high to low and a remainder. In this context, the low-frequency components mostly represent effective signals, while the high-frequency components mostly represent noise signals. Therefore, as the order of IMFs increases, the noise components contained within the IMFs gradually decrease, while the signal components increase. Based on the proportion of noise and signal components within IMFs, IMFs can be classified into noise IMFs, mixed IMFs, and signal IMFs.

In the classification of IMF component types, the Consecutive Mean Square Errors (CMSE) analysis is initially used to distinguish between noise IMFs and mixed IMFs. This method does not require any external reference information. In the classification between noise IMFs and mixed IMFs, two consecutive reconstructed signals are compared to assess their degree of similarity, as shown in the following equation:

(9) $$CMSE\left(\tilde{Z_{k}},\tilde{Z_{k+1}}\right)=\frac{1}{N}\sum_{i=1}^{K}{\left[\tilde{Z_{k+1}}(t_{i})-\tilde{Z_{k}}(t_{i})\right]}^{2}$$

Here, $\tilde{Z_{k}}=\sum_{m=k}^{K}V_{IMF}^{m}+r(n)$ represents the difference between two consecutive reconstructed signals $\tilde{z}$*_k_* and $\tilde{z}$*_k+1_*, which corresponds to the *k* order IMF.

Therefore, the magnitude of CMSE reflects the similarity between the two consecutive reconstructed signals and also indicates the amount of signal energy in each order of IMFs. As the order of IMFs increases, the CMSE values generally decrease firstly and then increase. The initial decrease in CMSE values is due to the lower-order IMFs containing almost no signal, while the noise component decreases with increasing order. The subsequent increase in CMSE values is attributed to the emergence of signal components in the IMFs, which gradually increase with higher orders. Therefore, taking the order of IMFs with the minimum CMSE value as the demarcation point M1 between noise IMFs and mixed IMFs, the following equation is satisfied:

(10) $$M_{1}=argmin[CMSE(\tilde{Z_{k}}-\tilde{Z_{k+1}})]$$

Then, the *L_2_* norm method is used to distinguish signal–noise mixed IMFs and signal IMFs, by calculating the distance between the probability density functions of each IMF and the original gyroscope signal. Given two sets of Probability Density Functions (PDFs) for the data, denoted as *P* and *Q*, the Frobenius norm calculation satisfies the following equation:

(11) $$D\left(i\right)=dist[PDF\left(x\left(t\right)\right),PDF({IMF}_{i}(t))]$$

In this method, the Frobenius norm approach measures the distance between each order of IMFs and the original gyroscope signal to measure the similarity between them. When the distance between the PDFs reaches its maximum value, it indicates that the current IMFs are most similar to the original gyroscope signal. Considering the presence of noise in the original signal, the order of IMFs that achieves the maximum value plus one is defined as the demarcation point M2 between mixed IMFs and signal IMFs, as shown in the following equation:

(12) $$M_{2}=argmax\left[D\left(i\right)\right]+1$$

After distinguishing IMFs into noise IMFs, mixed IMFs, and signal IMFs, different processing methods are applied to each type of IMF. An improved hybrid filtering algorithm with a new filtering structure is proposed. Threshold filtering is dynamically applied to noise IMFs to avoid the high-frequency signal loss issue caused by directly removing noise IMFs using conventional algorithms. For mixed IMFs, FLP filtering is applied to avoid excessive filtering caused by increasing thresholds. Finally, the noise IMF components processed through dynamic threshold filtering and the mixed IMF components processed through FLP filtering along with the signal IMF components and residual terms are reconstructed to obtain the signal after gyroscope noise reduction. The improved algorithm effectively eliminates outliers, reduces the gyroscope signal noise, and maximizes the retention of effective components in gyroscope signals. The reconstructed gyroscope signal achieves a higher signal-to-noise ratio, significantly enhancing the performance of inertial navigation. 

The hybrid filtering structure achieves a refined and adaptive processing of gyroscope signals by combining the Letts criterion, CEEMDAN decomposition, CMSE, and *L*_2_ norm methods. Compared with traditional EMD noise reduction and wavelet noise reduction methods, this hybrid filtering structure demonstrates superior performance in retaining the effective signal components, enhancing the noise reduction effect, and offering robust adaptability. It is highly suitable for signal processing in complex dynamic environments. This provides strong support for high-precision autonomous localization for trains in enclosed environments such as tunnels.

## 3. Construction of SINS/OD Integrated Navigation Model

In a tunnel environment, the GNSS signals are prone to interruptions due to obstruction. Therefore, it is essential to integrate pulse observation signals into the SINS/OD integrated navigation model mentioned above, which can compensate for rapid positioning accuracy degradation in enclosed environments. This section will elaborate on the construction of the SINS/OD integrated navigation system model, including the specific forms and mathematical expressions of the state equations and observation equations. This addresses the uncertainty and errors in navigation models based on pulse observations.

### 3.1. State Equation

The system constructs a model that includes various navigation error parameters, such as position error *δ*$p_{e}^{n}$, velocity error *δ*$\nu_{e}^{n}$, attitude error $\phi_{e}^{n}$, accelerometer bias ${▽}^{b}$, gyroscope constant drift $\varepsilon^{b}$, two SINS/OD installation error angles $\alpha_{\theta }$, odometer scale factor error *δk*, system noise *W_f_*, and $\alpha_{\Psi }$ as the system state variables. These variables form an 18-dimensional complex state space model, as shown in the following Equation (13) and the mathematical expression of the system state equation is shown as Equation (14):

(13) $$X={\left[\begin{array}{cccccccc} \phi_{e}^{n} & \delta v_{e}^{n} & \delta p_{e}^{n} & \varepsilon^{b} & \nabla^{b} & \delta k & \alpha_{\psi } & \alpha_{\theta } \end{array}\right]}^{T}$$

(14) $$\dot{X}=A_{s}X+W_{f}=\left[\begin{array}{cc} A_{INS} & 0_{3\times 3}\\ 0_{3\times 15} & 0_{3\times 3} \end{array}\right]+W_{f}$$

Among them, *X* represents the state variables, and *A_INS_* represents the state transition matrix composed of various navigation error parameters, which used to simulate the evolution of the navigation system state. This equation includes various error models, such as position error, velocity error, attitude error, and sensor zero bias [19].

### 3.2. Observation Equation

The observation equation is established based on velocity matching within the carrier system by comparing the odometer output speed with the speed output of the inertial navigation system. The key to this equation is handling and compensating for the installation errors and lever arm errors between the SINS and OD [20].

The core of the observation equation is to use the observation matrix *H* and state variables *X* to estimate the observation error. The difference between the velocity outputs of the SINS and the odometer is selected as the observation quantity. The mathematical expression is shown as follows:

(15) $$Z=\delta v_{\mathrm{ins}}^{n}-v_{D}^{n}\times \phi +C_{b}^{n}e_{3}v_{D}\alpha_{\theta }-C_{b}^{n}v_{D}\alpha_{\psi }-C_{b}^{n}v_{D}\delta k$$

(16) $$Z=HX+V_{f}$$

(17) $$H=\left[\begin{array}{ccccc} -v_{D}^{n}\times I_{3\times 3} & 0_{3\times 9} & C_{b}^{n}e_{3}v_{D} & -C_{b}^{n}e_{1}v_{D} & -C_{b}^{n}e_{2}v_{D} \end{array}\right]$$

While *Z* represents the observed quantity, *H* represents the observation matrix and *V* represents the observation noise. ${\hat{C}}_{b}^{n}$ is the estimated direction cosine matrix from the body frame to the navigation frame, which is obtained by applying a small correction to the actual direction cosine matrix ($C_{b}^{n}$). The transformation matrix from the inertial navigation coordinate system to the navigation coordinate system is given by the following equation:

(18) $${\hat{C}}_{b}^{n}=(I-\phi \times )C_{b}^{n}$$

**Figure 2:**
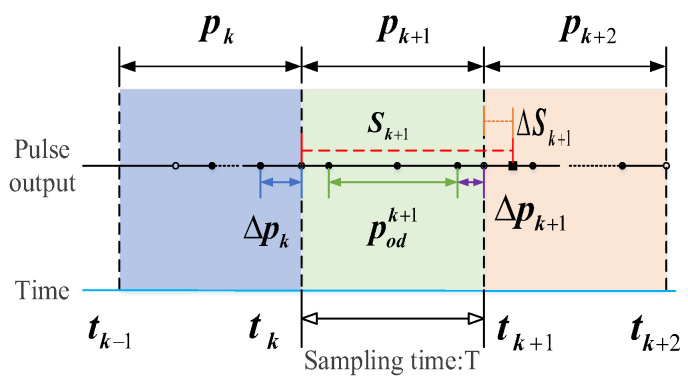
Schematic diagram of mileage meter pulse model.

The odometer pulse output is related to the vehicle’s motion state as shown in Figure 2. The number of odometer pulses generated per unit time is determined by the vehicle’s speed. Due to the high accuracy of the inertial navigation equipment, it can accurately reflect the vehicle’s motion state [21]. The conversion of inertial navigation output into pulse output is related to the true value of odometer pulses *P_k_*_+1_ as follows:

(19) $$P_{k+1}=\frac{S_{k+1}-\Delta S_{k+1}}{K}={\hat{p}}_{\mathrm{ins}}^{k+1}-\delta p_{\mathrm{ins}}^{k+1}$$

Considering the same sampling time interval (*t_k_*, *t_k_*_+1_), the number of pulses induced by vehicle inertia is represented by the following equation:

(20) $$\begin{array}{l} {\hat{p}}_{\mathrm{ins}}^{k+1}=\frac{S_{k+1}}{K}=\frac{1}{K}\left(v_{k}^{m}T+\frac{1}{2}a_{k+1}^{m}T^{2}\right)\\ =\frac{T}{2K}(v_{k}^{m}+v_{k+1}^{m})=\frac{T}{K}v_{k,k+1}^{m} \end{array}$$

In the formula, *S_k_*_+1_ represents the distance traveled by the vehicle during the sampling time, and *ν_k_* and *ν_k_*_+1_ denote the vehicle’s velocity in the m system at times *t_k_* and *t_k_*_+1_, respectively. $\nu_{k,k+1}^{m}$ is the mid-point velocity, and $a_{k+1}^{m}$ is the vehicle’s acceleration, representing the true value of the odometer pulse.

By employing the velocity matching method, we compare the odometer output observed velocity in the carrier system with the inertial navigation output velocity. Simultaneously, we combine the angular misalignment and velocity projection errors and the velocity of constructing an inertial navigation system as shown in the following equation:

(21) $$v_{obs}=v_{INS}+R\Delta \theta$$

where ∆*θ* is the angular misalignment, and *R* is the relational rotation matrix. Based on the above, the observation matrix in measurement equation *Z* = *HX* + *V* can be expressed as follows:

(22) $$H=\left[\begin{array}{ccccccc} 0_{3\times 3} & C_{b}^{n} & -C_{b}^{n}(v_{INS}^{n}\times ) & 0_{3\times 3} & 0 & 0 & v_{y}^{b}\\ 0_{3\times 3} & 0 & 0 & 0_{3\times 3} & 0 & -V_{OD} & 0\\ 0_{3\times 3} & -v_{y}^{b} & 0 & 0 & 0 & 0 & \left(\omega_{ib}^{b}\times \right) \end{array}\right]$$

where $C_{b}^{n}$ is the direction cosine matrix from the body frame to the navigation frame, $\nu_{INS}^{n}$ represents the INS velocity vector in the navigation frame, *ν_OD_* denotes the velocity measured by the odometer, $\nu_{y}^{b}$ is the velocity component in the *y*-direction of the body frame, and $W_{ib}^{b}$ is the angular velocity vector in the body frame.

Through detailed mathematical description and model construction, the dynamic behavior of the SINS/OD integrated navigation system is revealed. It also provides a thorough analysis of key error sources, laying the foundation for subsequent navigation accuracy improvement and system optimization.

## 4. Adaptive Kalman Filtering Algorithm Based on EM

### 4.1. Algorithm Introduction

Applying the adaptive Kalman filtering algorithm based on EM estimation to train autonomous positioning in a pulse-observed tunnel environment can form a new train positioning strategy [22]. This strategy effectively addresses the challenge of train positioning in environments such as tunnels where GNSS signals are limited.

Considering the Non-Line-Of-Sight (NLOS) and multipath effects in a tunnel environment that may cause biases in observational data, the EM algorithm can be used to model and estimate these influences. The E-step can be used to estimate hidden states (such as the true position of a train), while the M-step updates model parameters (such as compensation for multipath effects) to better adapt to changes in the environment.

In the E-step, the computational load increases significantly because, in each iteration, the conditional expectation of the hidden variables needs to be computed. This usually involves complex probability distribution calculations and high-dimensional integrals. In the M-step, the computational complexity is high because, based on the expectations obtained from the E-step, model parameters need to be updated to maximize the likelihood function, which requires complex numerical optimization techniques. Next, the optimized observation model and system model will be applied in the Kalman filter to continuously estimate the train’s position through prediction and update steps [23]. 

As the train moves through the tunnel, external conditions and internal states may continuously change, which requires the algorithm to self-adjust to adapt to these changes [24]. By dynamically adjusting the covariance of observation noise and process noise, the Kalman filter can better adapt to current observation conditions. At the same time, periodically reevaluating based on the EM algorithm and adjusting the error model under multipath effects and NLOS conditions ensures that the positioning system maintains a high accuracy throughout the entire tunnel travel process.

### 4.2. Flowchart

**Figure 3:**
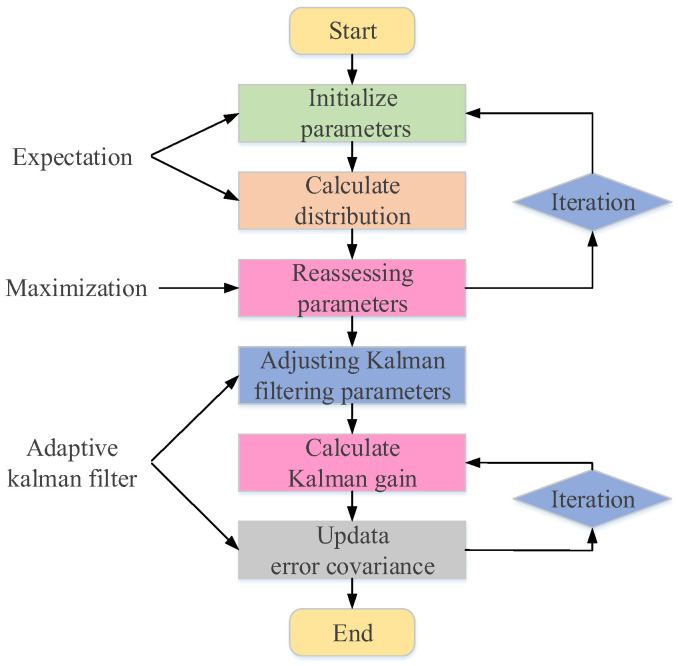
Algorithm flow chart.

The flowchart of the EM-based adaptive Kalman filtering algorithm, as shown in Figure 3, demonstrates how the algorithm alternates between predictions and updates in each iteration and dynamically adjusts the system model and Kalman filter parameters to achieve adaptive state estimation.

### 4.3. Integrated Algorithm

(1) Initialize parameters:

Set the initial state estimate as $\hat{X}\left\{0|-1\right\}$, initial error covariance as $P\left\{0|-1\right\}$, and initial parameter estimate as $\theta^{\left(0\right)}$.

(2) Combination of the EM algorithm:

E-step: Calculate the conditional expectation of the latent variables *Z* given the observed data *X* and the current parameter estimate $\theta^{\left(t\right)}$. This typically involves computing the posterior distribution of the latent variables, as shown in Equation (23):

(23) $$Q(\theta |\theta^{\left(t\right)})=E[\log P(X,Z|\theta )|X,\theta^{\left(t\right)}]$$

Here, log *P*(*X*, *Z*|*θ*) is the log-likelihood function of the complete data.

M-step: Update model parameters to maximize the log-likelihood function of the observed data and find the parameter *θ* that maximizes the *Q* (*θ*|$\theta^{\left(t\right)}$), as shown in Equation (24):

(24) $$\theta^{\left(t+1\right)}=\arg\max_{\theta }Q(\theta |\theta^{\left(t\right)})$$

Compare $\theta^{\left(t+1\right)}$ and $\theta^{\left(t\right)}$, or their corresponding log-likelihood values to determine if they meet a certain convergence criterion (the difference is less than a threshold). When the parameter estimation converges, the algorithm terminates and yields the estimated values of the parameters. Specifically, for the covariance matrices *Q_k_* and *R_k_* of process and observation noise in the Kalman filter, they can be adjusted and optimized using the EM algorithm in each iteration. The core of the EM algorithm lies in the alternating execution of the E-step and M-step, continuously using the expectations obtains from the E-step to optimize the parameter estimates in the M-step until the convergence criteria are met. This algorithm is particularly suitable for parameter estimation problems involving incomplete observed data or latent variables.

(3) Kalman filtering itself is an efficient recursive filter, but its computational complexity increases when dynamically adjusting the noise covariance matrix. At each time step, Kalman filtering requires two main steps: prediction and update. Although these steps themselves are relatively simple, in high-dimensional state spaces and complex observation models, the computational load of matrix operations can become quite large. The Kalman filter loop is similar to the method applied by Jiang et al. for correcting noise in dynamic mode decomposition [25].
(4) Iteration and convergence:

Repeatedly execute the E-step and M-step of the EM algorithm, as well as the Kalman filter cycle, until the algorithm converges or reaches the maximum number of iterations. In summary, while the CEEMDAN-EM-KF method improves the accuracy for the autonomous localization of trains in tunnel environments, it also introduces higher computational complexity. Finally, evaluate the positioning accuracy of the adaptive Kalman filtering algorithm based on pulse observation and EM estimation by testing it in a real or simulated tunnel driving environment. It is necessary to collect a large amount of data to analyze the algorithm’s compensation effects for multipath effects and NLOS errors, as well as its positioning accuracy under various driving conditions.

(5) Simulation verification and analysis:

To validate the effectiveness of the proposed method in the paper, simulation experiments were designed. The simulation parameters for the experiment are shown in Table 1.

**Figure 4:**
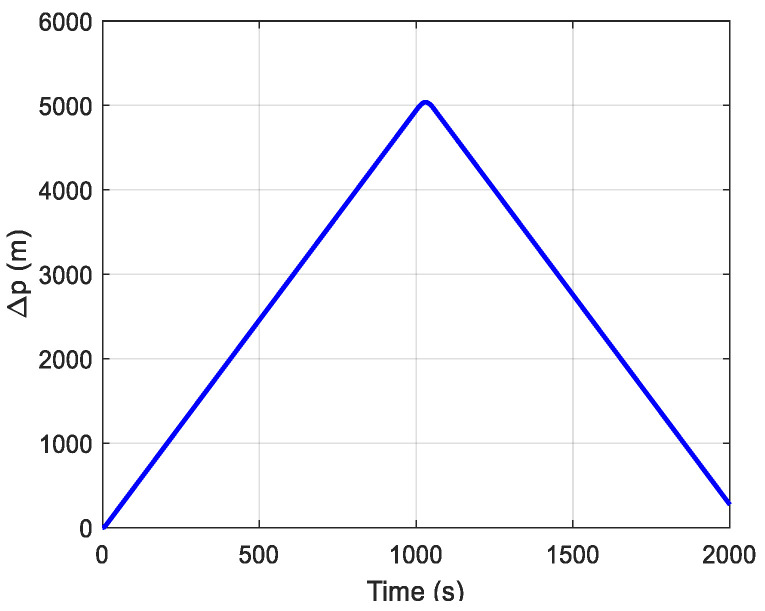
Simulated trajectory plot.

The simulation experiment involves the normal driving of a train inside a tunnel lasting a total of 2000 s. Meanwhile, considering the complexity of the train operating environment and in order to better simulate real-world conditions, noise interference is injected into the simulated train odometer measurement information. Additional noise and outlier information are added based on the simulation parameters, with the noise information as shown in the following equation:

(25) $$v_{R}∼\left\{\begin{array}{c} \begin{array}{cc} N\left(0,R_{R}\right) & w.p.0.40 \end{array}\\ \begin{array}{cc} N\left(0,5R_{R}\right) & w.p.0.60 \end{array} \end{array}\right.$$

(26) $$v_{A}∼\left\{\begin{array}{c} \begin{array}{cc} N\left(0,R_{A}\right) & w.p.0.40 \end{array}\\ \begin{array}{cc} N\left(0,5R_{A}\right) & w.p.0.60 \end{array} \end{array}\right.$$

where *ν_R_* and *ν_A_* represent the noise characteristics of slant range and azimuth, respectively. The *w.p.*0.60 represents the measurement noise with a variance of five times the original, and appears in the original Gaussian distribution with a probability of 60%. As shown in Figure 4, a simulated trajectory curve is set to simulate the train’s movement in a tunnel environment, with a sharp turn occurring at 1000 s to better explore the effectiveness of the method proposed in this paper for SINS/OD integrated positioning during satellite signal denial.

**Figure 5:**
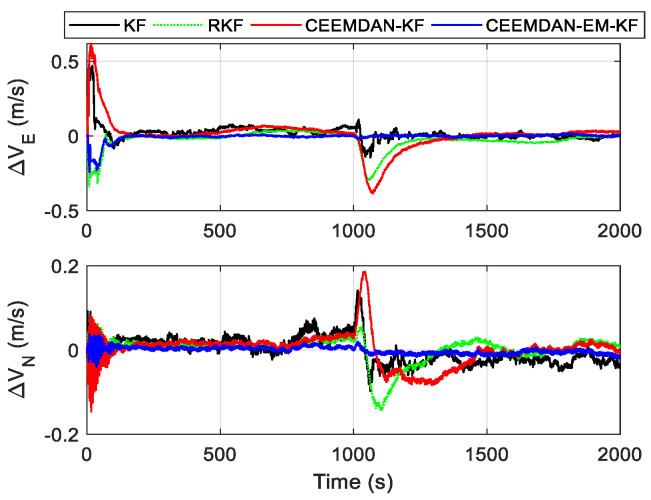
Velocity error curve.

Based on the simulated trajectory settings described above, a comparative validation of autonomous positioning methods for trains in tunnel environments is conducted, which includes traditional Kalman filtering SINS/OD integrated navigation methods and robust Kalman filter methods [26], as well as the combined positioning methods based on CEEMDAN denoising and CEEMDAN-EM-KF proposed in this paper. Based on the above simulation conditions and parameter settings, we first compared the eastward and northward velocity errors of each method as shown in Figure 5. It can be observed that all methods exhibit significant eastward and northward errors in the initial stages, which converge rapidly to smaller error ranges over a short period. However, at 1000 s when the train undergoes a turning maneuver, the eastward and northward velocity errors of the first three methods rapidly increase, failing to demonstrate effective suppression, resulting in significant drift in both eastward and northward velocities. In contrast, CEEMDAN-EM-KF shows stable performance, exhibiting good velocity tracking and matching during the turning maneuver.

**Figure 6:**
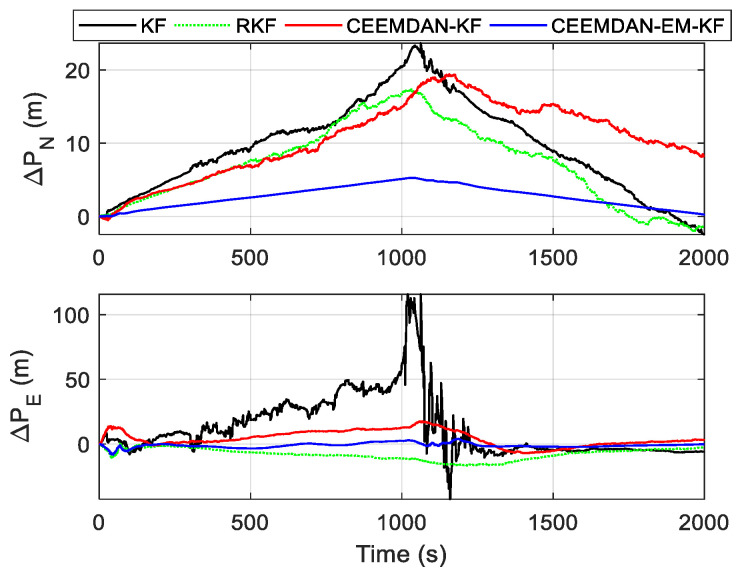
Chart of the position error curve.

In terms of positioning accuracy comparison, combined with Figure 6, it can be concluded that the magnitude of position error is generally proportional to the extent and duration of velocity drift. The CEEMDAN-EM-KF method exhibits the smallest velocity drift and demonstrates the lowest position error and highest position tracking accuracy. The maximum absolute values of eastward and northward position errors for each method are shown in Table 2, demonstrating the advantages and significant effectiveness of the method proposed in this paper in tunnel environments.

**Figure 7:**
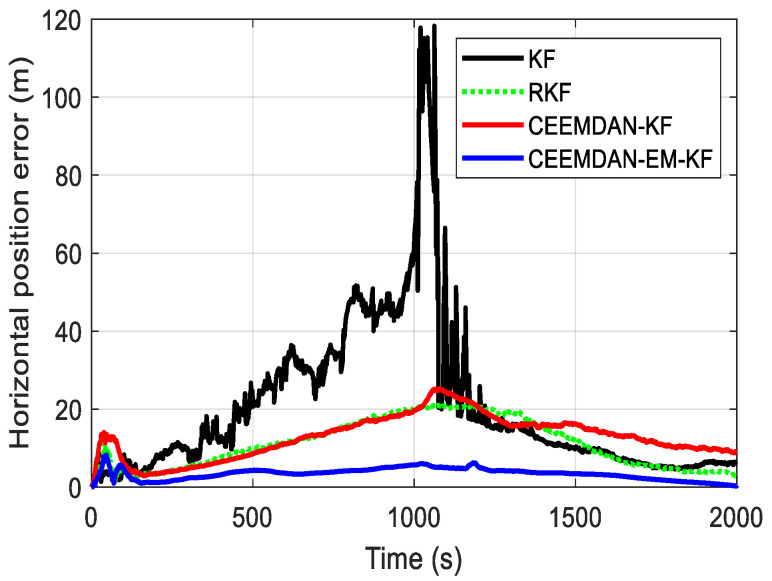
Drawing of the horizontal position error.

Finally, the performance of each method in horizontal position error was compared and analyzed. As shown in Figure 7, it can be observed that the traditional Kalman filtering SINS/OD integrated navigation method exhibits severe position divergence due to gyroscopic cumulative errors with a maximum horizontal error exceeding 100 m over the entire trajectory. The RKF and CEEMDAN-KF methods show comparable performance in horizontal position error, demonstrating effective suppression after improving the inertial unit performance, with maximum errors controlled around 20 m. In contrast, the CEEMDAN-EM-KF method reduces horizontal position error to less than 10 m, demonstrating the best performance among the methods compared and analyzed.

### 4.4. Discussion

An autonomous positioning method for trains based on pulse observation for tunnel environments is proposed in this paper to address the issue of insufficient positioning accuracy in enclosed environments such as tunnels and culverts. Compared with common train positioning methods (such as GNSS and INS), under the condition of poor GNSS signal, the error of the trajectory is relatively significant, while the accuracy of INS will decrease over time [27,28]. This method, by combining the EM algorithm with adaptive Kalman filtering, effectively addresses the challenges of Non-Line-of-Sight (NLOS) and multipath effects in tunnel environments. As shown in Figure 7 and Table 2, it significantly enhances the accuracy and robustness of train positioning. Additionally, the method exhibits strong adaptability. The adaptive Kalman filter dynamically adjusts the covariance matrices of process noise and observation noise, enabling the filter to maintain optimal performance across different environments and conditions. This adaptability enables the positioning system to continuously provide high-precision location information within tunnels. Finally, when constructing the SINS/OD integrated navigation model, an in-depth analysis of error sources was conducted, including installation errors and lever arm errors. These error sources were incorporated into the observation equations. This detailed error modeling allows the positioning algorithm to more accurately reflect the internal dynamic characteristics of the system, thereby further enhancing the positioning accuracy of the train.

However, this method also has some drawbacks. The combination of the EM algorithm and adaptive Kalman filtering, while improving positioning accuracy and robustness, also increases computational complexity. In train positioning systems where real-time performance is crucial, computational complexity could become a limiting factor. Especially in complex environments such as tunnels, where train positioning needs to be performed in real-time, the computational process of the EM algorithm and adaptive Kalman filtering can be time-consuming. In practical applications, further optimization of the algorithm structure may be necessary to improve computational efficiency.

## 5. Conclusions

This paper proposes a train autonomous localization method based on pulse observations in a tunnel environment. This study presented the following:

(1) This study proposed an improved hybrid filtering algorithm with a new filtering structure, and the improved algorithm effectively eliminates outliers, reduces the gyroscope signal noise, and maximizes the retention of effective components in gyroscope signals. The reconstructed gyroscope signal achieves a higher signal-to-noise ratio, significantly enhancing the performance of inertial navigation. This lays a solid foundation for continuous and reliable inertial navigation data for subsequent train combination navigation and autonomous positioning.
(2) Pulse observation signals were integrated into the SINS/OD integrated navigation model mentioned above and could compensate for rapid positioning accuracy degradation in enclosed environments. Through detailed mathematical description and model construction, the dynamic behavior of the SINS/OD integrated navigation system was revealed. This also provided a thorough analysis of key error sources, laying the foundation for subsequent navigation accuracy improvement and system optimization.
(3) In terms of localization accuracy, the magnitude of the position error is generally positively correlated with the degree of speed drift and the drift time. The CEEMDAN-EM-KF method results in the smallest position error with significantly improved position tracking accuracy compared to other methods. This shows that using EM estimation combined with the adaptive Kalman filter method can better enable the localization system to maintain continuous high-precision position information output in a tunnel environment.
(4) The train self-localization method based on pulse observations in tunnel environments, which employs EM estimation combined with adaptive Kalman filtering, also has some drawbacks. Firstly, the combination of the EM algorithm and adaptive Kalman filtering increases computational complexity, which may become a significant limiting factor. Secondly, the performance of adaptive Kalman filtering heavily depends on parameter settings. Adjusting these parameters requires extensive experience and a large amount of experimental data; otherwise, it may lead to a decline in localization performance. Finally, in complex environments like tunnels, train localization requires the processing of large amounts of data, and the computation involved in the EM algorithm and adaptive Kalman filtering can be time-consuming.

## Figures and Tables

**Table 1:** SINS/OD performance metrics.

Sensor	Output Frequency	Indicator	Unit	Parameters
Inertial Measurement Unit	100 Hz	Gyroscopic drift	°/h	0.05
		Accelerometer bias	µg	200
Odometer	1 Hz	Scale factor	/	0.004

**Table 2:** Maximum absolute position error values for different methods.

Approaches	Eastward Position Error (m)	Northward Position Error (m)
KF	115.7	23.3
RKF	16.2	17.3
CEEMDAN-KF	17.2	19.2
CEEMDAN-EM-KF	2.9	5.2

## Data Availability

Data are contained with the article.
